# Application of histological method for detection of unauthorized tissues in meat sausage

**DOI:** 10.30466/vrf.2018.89154.2160

**Published:** 2019-12-15

**Authors:** Arezo Moghtaderi, Ahmadreza Raji, Saied Khanzadi, Abolghasem Nabipour

**Affiliations:** 1 *DVSC Graduate, Department of Basic Sciences, Faculty of Veterinary Medicine, Ferdowsi University of Mashhad, Mashhad, Iran; *; 2 *Department of Basic Sciences, Faculty of Veterinary Medicine, Ferdowsi University of Mashhad, Mashhad, Iran; *; 3 *Department of Food Hygiene and Aquaculture, Faculty of Veterinary Medicine, Ferdowsi University of Mashhad, Mashhad, Iran.*

**Keywords:** Histology, Sausage, Unauthorized tissues

## Abstract

Nowadays, the consumption of meat and meat products has been increased with modern manufacturing techniques. Due to the economic value, the likelihood of using unauthorized tissue is possible in meat products. The aim of this study was to apply morphological methods for detection of unauthorized tissues in meat sausage. In this study, a total number of 20 samples of different types of sausages were randomly collected from markets, in north-east of Iran. Each sample was divided into three equal parts and three paraffin-embedded blocks were prepared from each part (180 blocks). Then the sections were stained using Hematoxylin and Eosin, Masson’s trichrome, Periodic acid- Schiff/Alcian blue and Verhoeffe/Van Gieson. A total number of 720 slides were observed using a light microscope. This research showed the use of unauthorized tissues in the sausages which was detected by histological methods. We observed authorized tissues like skeletal muscle fiber (100%), fat tissue (100%) and plant material (97.70%). A wide range of unauthorized tissues were detected including dense connective tissue (6.66%), cartilage (28.30%), bone (8.30%), skin (51.60%), smooth muscle (1.66%) and blood vessels (11.66%). The results of this study confirmed the use of unauthorized tissue in meat sausages in Iran and concluded that the histological methods especially Masson’s trichrome staining are a practical technique for routine assessment of authenticity and quality of sausage to protect the consumers from adulteration.

## Introduction

Meat is an important part of the human diet with strong implications in health, economy, and culture worldwide. Meat production involves numerous domestic species, depending on many factors like religious and cultural beliefs, convenience and availability.^1^ Due to the economic value, the likelihood of using unauthorized tissue is possible in meat products.

To date, numerous identification methods have been developed for the detection of meat species, with conventional methods, which include physical, sensory analysis, anatomical, histological, chemical, biochemical, chromatographic, spectrophotometric, electrophoretic, immune sera diffusion, immunological and immuno-electrophoretic techniques.^2^^,^^3^

To evaluate the composition of sausages, the histological method can be used. The histological method can precisely evaluate the quality parameters of sausages by detecting specific tissue of animal organs, connective tissues, fat content, bone tissue, and others.^4^

Based on Iranian National standard regulations, the use of undesirable organs of slaughtered animals, including the visceral organs, hyaline cartilage, skin, fat, and bone instead of meat in cooked meat products is considered as fraud.^5^ Previously, some researchers have reported histological methods, as a useful technique to detect unauthorized tissues in some meat products. ^6^^-^^8^ This study was designed to evaluate the accuracy of the routine and special histological staining as a simple and inexpensive method for the determination of unauthorized tissue in meat sausages in Mashhad, in the north-east of Iran.

## Materials and Methods

In this study, a total number of 20 meat sausage from different processing factories were randomly collected from supermarkets of Mashhad in north-east of Iran. The characteristics of the samples, including sample type and date of sampling were recorded and then the samples were immediately transported to the histology laboratory of Faculty of Veterinary Medicine, Ferdowsi University of Mashhad. Each sample was divided into three equal parts, and then, three pieces from each part were fixed in 10.00% neutral-buffered formalin and were embedded in paraffin and routinely processed for light microscopy (180 blocks).^9^ The paraffin-embedded blocks were cut into 6.00 µm sections and stained using hematoxylin and Eosin (H & E), Masson’s trichrome (MT), Verhoeff/Van Gieson, Periodic Acid Schiff (PAS)/Alcian blue (AB) for histological study. The slides were observed under a light microscope (CX-21; Olympus, Tokyo, Japan) with a digital camera (DP25; Olympus) at 40× magnification to identify the presence of various types of animal tissues. Totally, 720 slides were prepared.

## Results

**Fig. 1:**
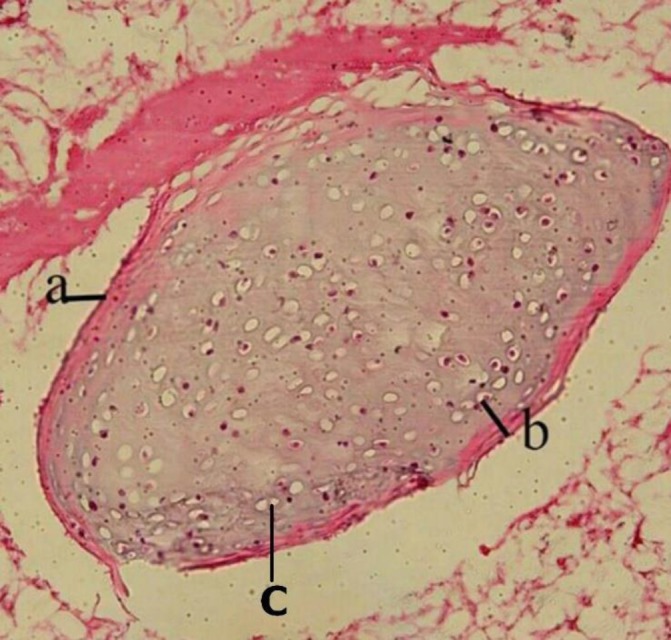
Photomicrograph of cartilage in the sausage. a: perichondrium, b: chondrocyte, and c: lacuna (H&E, 400×)

**Fig. 2:**
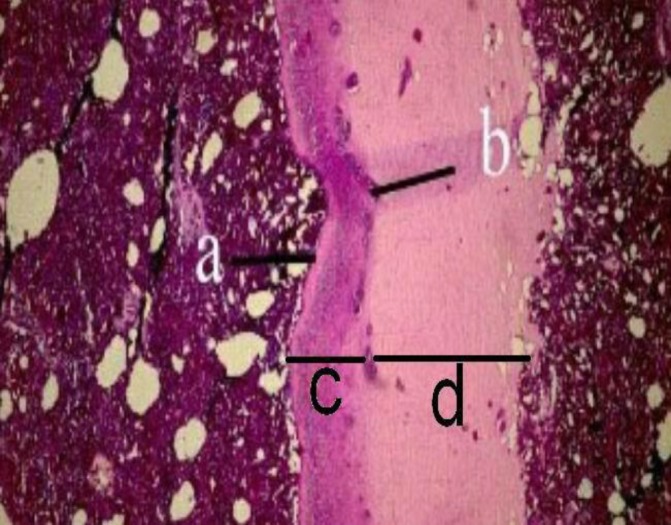
Photomicrograph of skin in the sausage. a: stratum corneum, b: vascular layer, c: epidermis, and d: dermis (PAS/AB, 100×)

 The histological technique may be a simple and economic tool for evaluation of the meat adulteration and to improve hygiene and meat quality. The present study showed the use of unauthorized tissue in the processed sausage products which were detectable by a histological method. We used six types of staining methods in order to detect unauthorized tissues in samples. In addition to skeletal muscles, light microscopy showed a variety of tissue types including dense connective tissue (6.66%), cartilage (28.30%; Fig. 1), bone (8.30%), skin (51.60%; Fig. 2), smooth muscle (1.66%), blood vessels (11.66%), adipose tissue (100%), and plant materials (97.70%). The percentages of various types of tissues detected in sausage by histological methods as well as best staining for detection of unauthorized tissue and results of staining are summarized in Table 1.

**Table 1:** Results of histological studies with various staining methods indicating different tissues in 180 samples of sausage

**Tissue**	**Staining**	**Results **	**Positive (%)**	**Negative**
**Skeletal muscle**	Verhoeff/van Gieson Masson’s trichrome	Muscle fiber: Yellow; Collagen fiber: Red Muscle fiber: Red; Collagen fiber: Green	180 (100%)	0
**Dense connective tissue**	Masson’s trichrome	Muscle fiber: Red; Collagen fiber: Green	12 (6.66%)	168
**Hyaline cartilage**	Masson’s trichrome	Matrix: Green; Chondrocyte: Red	51 (28.30%)	129
**Bone**	PAS/Alcian blue Masson’s trichrome	Compact bone: Dark purple; Spongy bone: Light purple Compact bone: Green; Spongy bone: Dark purple	15 (8.30%)	175
**Skin**	Verhoeff/van Gieson	Epidermis: Brown; Dermis: Pink	93 (51.60%)	87
**Smooth muscle**	Masson’s trichrome Verhoeff/van Gieson	Collagen fiber: Green; Muscle fiber: Red Collagen fiber: Red; Muscle fiber: Yellow	3 (1.66%)	177
**Blood vessels**	Verhoeff/van Gieson	Elastic fiber: Black; Smooth muscle: Yellow	21 (11.66%)	159
**Adipose tissue**	H&E	Fat: White	180 (100%)	0
**Plant materials**	PAS/Alcian blue	Plant cells: Green /Blue	176 (97.70%)	4

## Discussion

The unpermitted tissues identified in this study, using microscopic examination included dense connective tissue, hyaline cartilage, bone, skin, smooth muscle, and blood vessels. The use of unauthorized animal tissues in meat products is possible due to the economic value of meat itself. Authentications problem in meat products could include replacing meat species, tissue, vegetable proteins, organic compounds and also substitute vegetable fats with animal fats.^10^

 Histological methods could provide fraudulent and quality control of meat products for the government authorities.^10^ Baskaya *et al*. reported that cartilage tissue belonging to the alimentary canal in 3 of 27 ready-to-sell minced meat samples in Ankara.^11^ Cetin *et al*. reported that 21 out of the 127 ready to sell meat samples contained a high amount of calcium, suggesting the addition of non-meat materials, such as ground bone fragment and mechanically deboned meat.^12^ Latorre *et al.* showed the use of unauthorized tissues including connective tissue, gizzard, adipose tissue, soybean, cartilage, ovary, lymph node, and gland tissue in the Kabab Loghme (n = 5), sausage (n = 5), handmade hamburger (n = 5) and Kabab Koobideh (n = 5).^13^ The histological examination carried out in the USA on eight different brands of hamburgers and hotdogs showed the presence of connective tissue, blood vessels, peripheral nerve, adipose tissue, plant material, cartilage, and bone.^14^^,^^15^ Rokni *et al*. showed salivary gland tissue in the cooked sausage which reflects the use of meat obtained from the head of slaughtered animals in meat products.^16^ Sepehri Erayi observed additive tissue comprised of chicken skin, hyaline cartilage, peritoneal fat, and kidney in 30 samples from three different types of sausages.^17^ Sadeghi et al. examined 720 samples of sausages and revealed unauthorized tissues including adipose tissue (30.00%), cartilage and bone (96.20%), cardiac muscle(19.20%), immature bone (57.60%), spleen, aorta, esophagus, salivary gland, alimentary gland, lymphatic node, hair, lung and tongue (3.80%), breast, skin and nerve tissue (7.75%), connective tissue and smooth muscle (27.00%), and blood vessel (46.10%), in addition, herbal tissues was recognized in 100% of the examined samples.^3^

In this study, it is demonstrated that the MT staining was the best staining method for reorganization of unauthorized tissue in sausages which was in agreement with the findings of a previous study.^18^ Jahed Khaniki and Rokni showed a 10.00% presence of unpermitted tissue such as chicken gizzard, mammary glands, lung, head soft tissue and cattle visceral organs in the prepared sausage using MT staining.^18^ Sadeghinezhad *et al. *verified the efficacy of MT blue staining for detecting animal and herbal additive tissue in minced meat as well as common H&E staining.^19^ Pospiech *et al*. showed that some special stainings such as the PAS/ Calleja staining which targets polysaccharides, can indicate soybean flour.^20^

The present study showed the use of unauthorized tissue in sausages in north-east of Iran. This study was designed to evaluate the accuracy of the routine and special histological staining as a simple and inexpensive method for the determination of unauthorized tissue in sausages in north-east of Iran.
